# Lactylation modification - a bridge between sepsis and macrophage metabolic reprogramming

**DOI:** 10.3389/fimmu.2026.1738765

**Published:** 2026-03-06

**Authors:** Zhe Fang, Gui song Zhu, Deng Yun Nie, Biao Xu

**Affiliations:** Nanjing Hosptial of Chinese Medicine affiliated to Nanjing University of Chinese Medicine, Nanjing, China

**Keywords:** glycolysis, lactation modification, macrophages, metabolic reprogramming, sepsis

## Abstract

Sepsis is a life-threatening organ failure syndrome triggered by dysregulated host responses to infection. During disease progression, macrophages drive initial hyperinflammatory responses and critically regulate the subsequent immunosuppressive phase. Emerging evidence reveals that macrophage phenotypes dynamically adapt through metabolic reprogramming, creating phenotype-metabolism interdependence. Notably, lactate—long considered merely a glycolytic byproduct—now emerges as a key regulator through histone lactylation. This epigenetic modification fine-tunes macrophage functionality and corrects inflammatory imbalance in sepsis. This breakthrough illuminates lactylation’s central role in macrophage regulation, opening new diagnostic and therapeutic avenues. This review comprehensively examines lactylation mechanisms and their impact on metabolic control in sepsis-associated macrophages.

## 1 Introduction

Sepsis is an infection-induced syndrome of immune dysregulation, characterized by systemic inflammation that may progress to septic shock and fatal organ failure (1, 2). It accounts for 19.7% of global mortality (3), with in-hospital fatality rates reaching 25-30% (4, 5). Among septic shock patients, mortality risks escalate to 40-60% during hospitalization (6–8). Persistent immunosuppression critically worsens prognosis by increasing susceptibility to secondary infections (9). Within this process, phenotypically plastic macrophages serve as pivotal regulators bridging innate and adaptive immunity (10). Crucially, lactate—a central metabolite in carbohydrate and amino acid metabolism—holds dual clinical significance in sepsis: Zhang’s 2019 breakthrough on lactate metabolism (11) advanced metabolic reprogramming research while establishing lactate’s prognostic value, as elevated serum levels strongly correlate with mortality (12). Consequently, sustained post-resuscitation lactate >2mmol/L defines septic shock in Sepsis-3 guidelines (2).

Studies have shown that there is a certain correlation between lactate levels and lactoylation levels.

Recently discovered histone lactylation—a novel epigenetic modification—establishes direct links between lactate metabolism and immune responses in sepsis. Whereas histone lactylation (e.g., H3K18la) constitutes an epigenetic mechanism directly modulating chromatin states, non-histone lactylation primarily reprograms metabolic enzyme activity—creating dual regulatory axes. Deciphering lactylation’s regulatory mechanisms offers new diagnostic and therapeutic opportunities.

## 2 Sepsis and macrophages

### 2.1 Macrophage polarization in sepsis

Macrophages serve pivotal roles in innate immunity and antigen presentation (13). Their remarkable plasticity enables them to detect environmental threats, initiate immune responses, and undergo stimulus-specific polarization to modulate host immunity, generating diverse functional states (14, 15). Consequently, macrophages critically regulate immune homeostasis and inflammatory responses during sepsis progression.Functionally, macrophages polarize into two primary subtypes: classically activated (M1) and alternatively activated (M2). During initial infection, pro-inflammatory stimuli (e.g., IFN-γ and LPS) drive M1 polarization, amplifying inflammatory mediator production (including IL-1β, TNF-α, IL-6, and ROS) that precipitates cytokine storm. As sepsis advances, Th2 cytokines (IL-4, IL-13), TGF-β, IL-10, glucocorticoids, and immune complexes induce M2 expansion. This population secretes immunosuppressive factors (e.g., IL-10, TGF-β) that establish persistent immunosuppression (10).

### 2.2 Macrophage metabolic reprogramming in sepsis

Metabolic reprogramming represents a fundamental adaptive mechanism enabling cells to restore homeostasis by remodeling energy pathways and metabolite profiles in response to environmental cues.Core metabolic pathways include glycolysis, tricarboxylic acid (TCA) cycle, fatty acid oxidation, and amino acid metabolism (16). During sepsis, immune cells preferentially employ glycolysis over oxidative phosphorylation (OXPHOS) to rapidly fuel proliferation, differentiation, and vigorous inflammatory responses for pathogen clearance (17). This glycolytic shift also occurs in parenchymal cells, including renal tubular epithelial cells (TECs) (18) and cardiomyocytes (19). Under physiological conditions, macrophages primarily utilize glucose-fueled OXPHOS to meet energy demands (20). Sepsis triggers macrophage metabolic reprogramming characterized by enhanced glycolysis and suppressed mitochondrial oxidation. Lipopolysaccharide (LPS) induces protein s-nitrosylation in electron transport chain complexes (I and cytochrome c oxidase), simultaneously boosting glycolysis and impairing mitochondrial respiration (21, 22). Concurrent mTOR-HIF-1α activation by LPS upregulates key glycolytic enzymes—including GLUT1, PFKFB3, HK2, PKM2, and LDH—further amplifying glycolytic flux (23). Though AMPK normally promotes fatty acid β-oxidation via PPAR-γ/CPT1 regulation, its downregulation in sepsis enhances macrophage glycolysis while directly modulating IL-1β and IL-6 secretion (24). LPS-drived GLUT1 upregulation accelerates glucose uptake and glycolytic flux, stimulating lactate production that reinforces M1-like polarization (25). Mechanistically, PPP flux generates NADPH through glucose-6-phosphate dehydrogenase (G6PD) and 6-phosphogluconate dehydrogenase (6PGD). This NADPH pool fuels three critical pro-inflammatory processes: 1) NADPH oxidase 2 (NOX2) activation, producing microbicidal superoxide that amplifies NF-κB signaling via ROS-dependent MAPK phosphorylation; 2) glutathione reductase-mediated maintenance of reduced glutathione (GSH), protecting M1 macrophages from oxidative damage during respiratory burst; 3) *de novo* fatty acid synthesis via FASN, supporting inflammatory lipid mediator synthesis and membrane remodeling. Thus, PPP-derived NADPH serves as a metabolic linchpin coordinating oxidative burst, redox balance, and biosynthetic demands in classically activated macrophages (26, 27). M2-like macrophages inherently express elevated CARKL, which catalyzes sedoheptulose-7-phosphate formation to reduce PPP activity, GSH synthesis, and pro-inflammatory cytokine expression (28).

## 3 Lactic acid and sepsis

### 3.1 Lactic acid in sepsis

Lactate, the terminal metabolite of glycolysis, is ubiquitously present in physiological systems.Sepsis elevates lactate levels through multifaceted mechanisms. While traditionally attributed to anaerobic conditions, emerging evidence implicates accelerated aerobic glycolysis as a major contributor.During hyperinflammation, oxygen demand by activated immune cells induces tissue hypoxia, stabilizing hypoxia-inducible factor 1α (HIF1α) (29–31). HIF-1α orchestrates glycolytic reprogramming:It enhances glycolytic flux by upregulating glycolytic enzymes and glucose transporters (32, 33). Crucially, HIF-1α sustains ATP production under hypoxia by inducing LDH-A and glucose transporters (34, 35). It also mediates LPS-induced glycolysis in dendritic cells (36). Essential HIF-1α-dependent lactate production is documented in bone marrow neutrophils (37). Thus, HIF-1α-mediated lactate generation represents an adaptive response to inflammatory metabolic demands. This metabolic rewiring occurs irrespective of oxygen tension, with lactate serving as a co-regulator of immune function. Early sepsis also features proinflammatory cytokines where IL-1β critically drives aerobic glycolysis and lactate generation (38–41). TLR4-induced PKM2 tetramerization amplifies IL-1β transcription, potentiating lactate production in LPS-stimulated macrophages (42). Multiple cytokines (IL-2, IL-3, IL-7, IFN-γ, TNF-α) similarly enhance glycolytic lactate output (39–41). Collectively, these findings establish lactate as an inflammation biomarker rather than merely a hypoxia indicator (43). Clinically, serum lactate serves as a vital indicator of sepsis-induced organ dysfunction.While acute metabolic perturbations may delay lactate fluctuations, serial measurements remain prognostically significant: Persistent hyperlactatemia predicts mortality, whereas lactate clearance correlates with improved outcomes (44). Reduced lactate levels confer survival benefits across critical illnesses beyond sepsis, validating lactate kinetics as universal prognostic markers irrespective of initial values (45). Beyond its metabolic functions as fuel source and gluconeogenesis precursor, lactate acts as a signaling molecule through autocrine/paracrine/endocrine mechanisms to regulate systemic homeostasis.

### 3.2 Lactate-mediated lactylation modification

Lysine lactylation (Kla) was first identified as a histone post-translational modification at conserved lysine residues.In a landmark study, Zhang et al. (11) established lactate-induced lactylation of histone H3 at lysine 18 (H3K18la) as an epigenetic regulatory mechanism.Using HPLC-MS/MS and pan-Kla antibody immunoblotting, they demonstrated dose-dependent increases in histone lactylation upon exogenous L-lactate stimulation.Metabolic labeling with 13C3-L-lactate confirmed direct lactate incorporation into lysine residues.U-13C6-glucose tracing further verified endogenous glycolysis as the primary lactate source for this modification.Enhanced glycolytic flux relative to TCA cycle activity elevates cellular lactate, correlating with amplified lactylation.Rotenone-induced mitochondrial inhibition increased both intracellular lactate and histone lactylation. Conversely, glycolytic blockade by 2-deoxy-D-glucose (2-DG) substantially suppressed lactylation. Collectively, these findings define histone lactylation as a lactate-derived PTM dynamically regulated by cellular glucose metabolism.

### 3.2.1 Histone lactylation modification

**Figure 1:**
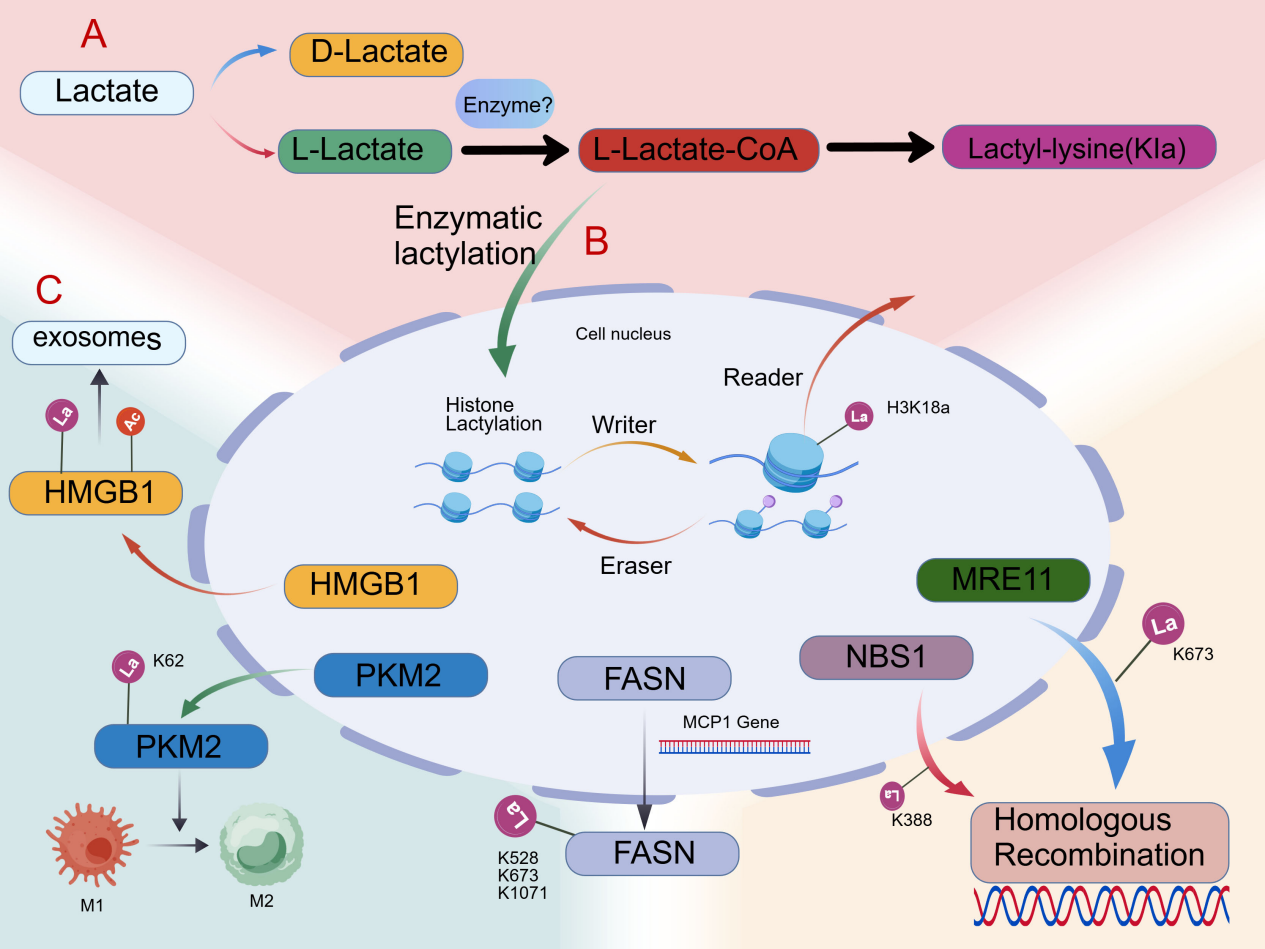
Structural and functional impacts of lactylation modifications. **(A)** Lactylation biochemistry **(B)** Functional Basis of Histone lactylation **(C)** Pathalogical signnificance of Non-histone lactylation.

Core histones (H2A, H2B, H3, H4) form evolutionarily conserved nucleosomes, with their N-terminal tails undergoing covalent modifications including methylation, acetylation, and phosphorylation, etc. These alterations remodel histone-DNA/histone-histone interactions, dictating chromatin condensation states to regulate gene transcription dynamics (46, 47). Lysine lactylation represents a novel post-translational modification (PTM) involving covalent lactate conjugation to lysine residues (**Figure 1A**). In histones, this modification remodels nucleosome architecture to regulate transcription (**Figure 1B**), while lactylation of functional proteins like PKM2 and HMGB1 drives metabolic reprogramming and inflammation (**Figure 1C**). This modification modulates protein functionality, stability, subcellular localization, and interactomes, thereby influencing diverse signaling pathways and biological processes. The lactylation machinery comprises four interconnected components:1. Lactate activation: Cellular lactate undergoes conversion to lactoyl-CoA. The precise enzymes mediating this step remain undefined.2. Writer: Nuclear lactoyl-CoA serves as the lactyl donor. Putative lactyltransferases catalyze covalent transfer to specific histone lysines.3. Reader: Specialized reader proteins detect lactylation marks, initiating downstream transcriptional programs.4. Eraser: Dedicated erasers catalyze delactylation to terminate signaling. This promotes chromatin condensation and RNA polymerase eviction, ultimately repressing gene expression (48).

Beyond its metabolic roles, histone lactylation regulates critical pathways:1. Fibrosis regulation:Cui et al. (49) demonstrated that histone lactylation boosts macrophage profibrotic polarization in pulmonary myofibroblasts.2. Phenotypic switching:TLR-BCAP activates PI3K-AKT-lactylation cascade (50), Drives macrophage transition from inflammatory to reparative states.3. IL-6/STAT3 axis: Lactylation directly activates Arg1 during LPS stimulation independent of M2 markers (51), Requires IL-6 autocrine/paracrine signaling via STAT3 phosphorylation.4. Epitranscriptome crosstalk: H3K18la mediates functional regulation of m6A reader YTHDF2 in uveal melanoma (52).

#### 3.2.2 Non-histone lactylation modification

Technological advances have expanded lactylation mapping beyond histones to functional non-histone proteins. In macrophages, non-histone lactylation reprograms metabolism and immune phenotypes by modulating glycolytic enzymes. Gaffney et al. (53, 54) identified non-enzymatic D-lactylation mediated by L-glutathione that inhibits glycolytic enzymes, establishing metabolic feedback. Lactylation at PKM2-K62 promotes tetramer stabilization, suppresses nuclear dimer translocation, and curtails lactate production to drive M1-to-M2 transition (55). Conserved ALDOA lactylation (56) suggests broader glycolytic regulation via this modification. Extracellular lactate uptake induces HMGB1 lactylation in macrophages, and co-modified (lactylated/acetylated) HMGB1 exacerbates sepsis via exosome-mediated inflammatory cascades (57, 58). MPC1 deletion elevates lactylation at three FASN lysines (K528/K673/K1071), with K673 critically regulating enzymatic activity (59). Lactylation dynamically modulates diverse functional proteins:1.AK2: Lactylation at K28 under hyperlactatemia alters enzymatic function (60). 2.DNA repair: MRE11-K673la enhances homologous recombination (HR), conferring chemoresistance (61). Complementary NBS1-K388 lactylation promotes HR-mediated repair (62). 3.Oncogenesis: p300-mediated NCL-K477la upregulates MADD via RNA splicing, activating MAPK signaling in cholangiocarcinoma (63).

### 3.3 Lactylation and organ dysfunction in sepsis

Lactylation directly fuels multiorgan failure in sepsis through cell-type-specific mechanisms:1.Renal injury: Lactylation of pyruvate kinase M2 (PKM2-K62la) in tubular epithelial cells sustains glycolytic flux but suppresses mitochondrial respiration, driving ATP depletion and acute kidney injury (55). 2.Cardiac depression: Extracellular vesicles carrying lactylated/acetylated HMGB1 from macrophages induce cardiomyocyte IL-1β overexpression, impairing contractility (57). 3.Hepatic steatosis: MPC1 deficiency-induced lactylation at FASN-K673 disrupts fatty acid oxidation, promoting lipid accumulation and hepatocyte death (59). 4.Pulmonary fibrosis: Histone H3K18la in alveolar macrophages upregulates profibrotic genes (e.g., TGF-β1), accelerating collagen deposition (49).

### 3.4 Bilateral regulation of lactation and glycolysis

**Figure 2:**
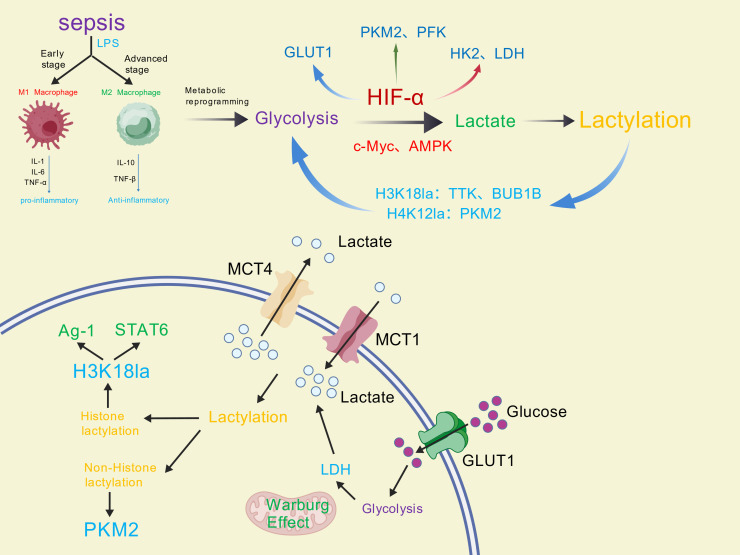
Bidirectional regulatory mechanism of lactation and glycolysis of macrophages in sepsis.

Lactylation and glycolysis exhibit mutually reinforcing interactions that critically influence diverse biological processes(**Figure 2**). Glycolysis accelerates lactate production—the essential precursor for lactylation—establishing a positive correlation between glycolytic flux and lysine lactylation levels (64).As a post-translational modification, lactylation structurally and functionally reprograms glycolytic enzymes to modulate metabolic pathway activity.

Glycolysis regulates lactylation by modulating lactate production through key mediators including HIF-α, c-Myc, and AMPK. In pulmonary hypertension, HIF-1α upregulates PDK1/PDK2 to enhance lactate production and elevate H3K18la/H4K5la lactylation levels, thereby stimulating pulmonary artery smooth muscle cell proliferation (65). Furthermore, HIF-1α binds promoter regions of glycolytic genes (e.g., GLUT1, HK, LDH, PKM2, PFK) to amplify their transcriptional activation and enzymatic activity (30, 66). The proto-oncogene c-Myc directly enhances aerobic glycolysis through transcriptional regulation of glycolytic genes (67). Chen et al. demonstrated that lactylation stabilizes NUSAP1 by inhibiting its proteolysis, establishing a NUSAP1-LDHA-glycolysis-lactate feedforward loop (68). Mechanistically, NUSAP1 forms a transcriptional complex with c-Myc and HIF-1α that upregulates LDHA expression to potentiate glycolysis and lactate generation. AMPK serves as a metabolic sensor maintaining cellular energy homeostasis. Importantly, AMPK suppresses aerobic glycolysis (Warburg effect) in cancer cells and inhibits tumor growth *in vivo* (69).

Lactylation influences glycolysis primarily by modifying transcription factors and structural components of key metabolic enzymes.In pancreatic ductal adenocarcinoma (PDAC), Li et al. (70) demonstrated that histone lactylation (particularly H3K18la) promotes cellular proliferation and migration by enhancing TTK and BUB1B transcription. These proteins subsequently increase P300 expression to amplify glycolysis. Furthermore, TTK phosphorylates and activates LDHA at tyrosine 239 (Y239), increasing lactate and H3K18la levels. This establishes a self-reinforcing loop connecting glycolysis, H3K18la, and TTK/BUB1B activity. In Alzheimer’s disease (AD), pro-inflammatory microglial activation shifts energy production from oxidative phosphorylation to glycolysis. H4K12 lactylation accumulates at promoters of glycolytic genes to activate transcription, creating a glycolysis/H4K12la/PKM2 positive feedback loop (71). Additionally, lactate in pro-inflammatory macrophages drives lactylation at position K26 of pyruvate kinase M2 (PKM2). This modification enhances its enzymatic activity, stabilizes its tetrameric form, and limits nuclear translocation - collectively reducing glycolytic reprogramming while promoting a pro-reparative macrophage phenotype (55).

## 4 Lactylation and macrophages

### 4.1 Lactylation regulates macrophage phenotypic transformation

Zhang et al. (11) identified an intrinsic “lactate clock” in M1 macrophages that coordinates their transition toward M2-like characteristics during late polarization phases via histone lactylation.

This regulatory mechanism links increased aerobic glycolytic flux during M1 polarization to elevated histone lactylation (Kla) and diminished histone acetylation (Kac).Histone Kla selectively promotes late-phase expression of M2-associated homeostatic genes (e.g., Arg1), while leaving early pro-inflammatory genes unaffected (11). As previously established, pro-inflammatory stimuli shift macrophage metabolism toward glycolysis while reducing oxidative phosphorylation (OXPHOS) dependence – mirroring the Warburg effect observed in cancer. Key metabolites such as lactate accumulate intracellularly and modulate diverse aspects of macrophage behavior. Accumulating evidence indicates lactate-mediated lactylation of both histone and non-histone proteins governs macrophage phenotypic plasticity. Noe et al. proposed that glycolytically-derived lactate initiates M1-to-M2 transitions by lactylating histone targets in early-phase M1 macrophages exhibiting high glycolytic/low TCA cycle activity (72). MCT4-mediated lactate efflux dynamically regulates histone lactylation during M1-to-M2 conversion, ultimately driving acquisition of a pro-reparative phenotype (73). Wang et al. demonstrated that PKM2 lactylation at K62 in M1 macrophages attenuates the Warburg effect and reduces lactate production, thereby accelerating conversion to an M2 reparative state (55).

### 4.2 Lactylation regulates macrophage metabolic reprogramming

In macrophages, pro-inflammatory signals (e.g., LPS and hypoxia) induce HIF-1α transcription via NF-κB activation. Newly synthesized HIF-1α translocates to the nucleus, heterodimerizes with HIF-1β, and binds hypoxia response elements (HREs) in target gene promoters. Concurrently, inactive dimeric PKM2 undergoes nuclear translocation and functionally interacts with HIF-1α. This complex coordinately drives expression of pro-inflammatory genes, promoting M1 macrophage polarization that amplifies pathogen responses and initiates inflammatory cascades (74–77). M1 macrophages enhance aerobic glycolysis through three primary mechanisms: First, nitric oxide (NO) produced by iNOS-mediated arginine catabolism impairs mitochondrial oxidative phosphorylation (OXPHOS), redirecting energy production to glycolysis (78). Second, TLR signaling via BCAP adapter proteins activates PI3K/AKT to augment glycolytic flux (79). Third, LPS-induced mitochondrial fragmentation reduces pyruvate dehydrogenase (PDH) activity and acetyl-CoA production, triggering compensatory glycolytic upregulation (80). Progressive lactate accumulation from glycolysis initiates an intrinsic “lactate clock”. Histone lactylation at H3K18 subsequently activates expression of M2-associated genes (e.g., Arg1) and their master regulator STAT6, ultimately driving transition to an anti-inflammatory M2 phenotype (11, 78). Notably, non-histone lactylation provides negative feedback to glycolysis: Lactylation of glycolytic enzymes inhibits their catalytic activity (54), while PKM2 lactylation enhances kinetic activity but prevents nuclear translocation—collectively reducing glycolytic output (55).

In sepsis, macrophages predominantly polarize toward the M1 state with enhanced glycolytic metabolism. Resultant lactate accumulation promotes both histone and non-histone lactylation, establishing bidirectional crosstalk between glycolysis and lactylation. This lactylation-mediated regulation promotes macrophage transition toward pro-reparative phenotypes, thereby fine-tuning the dynamic balance between inflammatory injury and anti-inflammatory resolution in sepsis pathogenesis.

## 5 Conclusion and prospects

Lactylation represents a newly identified post-translational modification initially discovered in macrophage metabolic studies. This lactate-driven modification, directly mediated by the glycolytic end-product lactate, establishes a novel regulatory link between immunometabolic states and phenotypic polarization. Given its central role in macrophage metabolic reprogramming, lactylation presents significant therapeutic potential. Targeting lactylation-associated enzymes, genes, or signaling pathways to redirect macrophage metabolism—disrupting immunosuppressive functions while promoting M2 polarization—may yield innovative sepsis treatments. Emerging evidence implicates lactylation dynamics as a predictor of sepsis progression. Animal studies show that delayed H3K18la accumulation in macrophages correlates with persistent inflammation and multi-organ injury (11). Elevated plasma lactylated HMGB1 levels in patients predict hepatic/renal dysfunction (57). However, clinical validation remains limited by: 1) Lack of standardized lactylation detection methods in human tissues; 2) Uncertain causality between lactylation thresholds and organ failure onset. Future studies must develop PET-based lactate metabolic imaging or lactylome profiling of circulating monocytes to enable real-time monitoring.

Beyond sepsis, lactylation serves as a conserved mechanism regulating macrophage function in diverse infections. In tuberculosis, lactate-induced histone lactylation reprograms macrophages toward a profibrotic M2-like phenotype, facilitating bacterial persistence (49). During SARS-CoV-2 infection, glycolytic monocytes exhibit elevated lactate flux, suggesting lactylation may modulate viral clearance (74). Localized infections (e.g., fungal keratitis) similarly show lactate-driven histone modifications that balance inflammatory resolution (73). Critically, sepsis represents a unique systemic scenario where lactylation’s dual regulation of inflammation and metabolism becomes clinically decisive due to organ-specific vulnerabilities.

Although lactylation is recognized as a crucial metabolic-epigenetic nexus in septic macrophages, current research remains exploratory, with scarce clinical data. Critical unresolved questions include: Does lactate accumulation always trigger lactylation? What nuclear lactate concentration threshold induces histone lactylation? Current studies predominantly use *in vitro* systems or simplified murine models, which may not fully recapitulate human pathophysiology. More sophisticated animal models that better mimic human physiology are urgently needed. Fundamental unknowns persist regarding: 1) Enzymatic regulation of histone lactylation (writers, readers, erasers), 2) Its crosstalk with histone acetylation, 3) Site-specific functional consequences, and 4) Associated receptors and signaling pathways.

Additionally, whether non-lysine residues (e.g., arginine, serine) undergo lactylation remains undetermined. Future research should prioritize: developing pathophysiologically relevant microenvironment models, systematically identifying modifying/de-modifying enzymes, and creating site-specific editing tools. These advances will facilitate novel sepsis therapies to improve clinical outcomes.
